# The impact of curated educational videos on pathology health literacy for patients with a pancreatic, colorectal, or prostate cancer diagnosis

**DOI:** 10.1016/j.acpath.2022.100038

**Published:** 2022-08-06

**Authors:** Ashish T. Khanchandani, Michael C. Larkins, Ann M. Tooley, David B. Meyer, Vijay Chaudhary, John T. Fallon

**Affiliations:** a Brody School of Medicine, East Carolina University, Greenville, NC, USA; b Medical Oncology Department, Vidant Medical Center, Greenville, NC, USA; c Department of Pathology and Laboratory Medicine, Brody School of Medicine, East Carolina University, Greenville, NC, USA

**Keywords:** Pathology report, Pancreatic cancer, Prostate cancer, Colorectal cancer, Health literacy

## Abstract

Despite patients having increased access to their own electronic health record (EHR) in recent times, patients are often still not considered a primary audience of pathology reports. An alternative to in-person patient education is the use of multimedia programming to enhance health literacy. Curated video presentations designed to explain diagnosis-specific pathology terms were reviewed by a board-certified pathologist and oncologist team and then shown to patients with a primary diagnosis of either pancreatic, colorectal, or prostate cancer in-clinic; these patients then completed a secure electronic survey immediately afterwards. Seventy patients were surveyed, with 91% agreeing or strongly agreeing that the video they watched increased their understanding of the medical terms used in their pathology reports, with a corresponding average Likert score (ALS) of 4.21 (SD = 0.77, CI = ± 0.18). Furthermore, 95% agreed or strongly agreed that the video they watched both enhanced their understanding of the role of the pathologist in diagnosing cancer (ALS = 4.27; SD = 0.65, CI = ± 0.15) and reported they found the video useful (ALS = 4.27; SD = 0.53, CI = ± 0.13). Curated videos such as those utilized in this study have the potential to increase patient health literacy and inform patients of the multidisciplinary nature of cancer diagnosis.

## Introduction

Over the past 10–15 years, the use of EHR has expanded dramatically; even more recently, patients have gained access to view their medical records, to include their own charts. The 2009 Health Information Technology for Economic and Clinical Health (HITECH) Act, as part of the American Reinvestment and Recovery Act, promoted the meaningful use of EHR based on defined health outcome priorities, two of which are the engagement of patients in their health and the improvement of health outcomes.^1^ Additionally, the American Medical Association (AMA) recognizes limited patient literacy as a barrier to effective medical diagnosis and treatment.^2^ Patients who are more involved in their own care may display better outcomes, an idea that has been explored with a host of diagnoses ranging from heart failure to diabetes, however challenges remain in the implementation of this communication, specifically concerning pathology reports.^3^^,^^4^ Despite patients having increased access to their own EHR, patients are often still not considered a primary audience of pathology reports; this reality may result in patients not understanding their own health reports.^5^^,^^6^ One survey of 129 oncologists at the Stanford Cancer Center in 2015 reports approximately 50% of those surveyed cited that the release of radiology and pathology reports to patients negatively affected communication with patients and many expressed a concern that patients would incorrectly interpret results.^7^ This concern is supported by current data: though the AMA recommends that written health materials not exceed a middle school reading level.^8^ One study found that many urologic pathology reports are written at an upper high school level.^8^^,^^9^ This implication is especially problematic considering many adults in the United States have limited literacy levels and that average health literacy may be even lower in vulnerable groups such as the impoverished.^8^^,^^10^ Furthermore, patients who cannot derive satisfactory information from their health records may seek explanatory information from sources such as the Internet. Unfortunately, these sources may provide incorrect information or, as one study found, are also written using terminology that is not well understood.^11^ This deficit in effective communication between healthcare providers and patients in medical reports creates an opportunity for pathologists to enhance patient understanding of pathology reports. While at least one effort has centered on providing in-person educational counseling, this process is not feasible in every situation due to time constraints and a lack of system of reimbursement for this service.^12^ As such, an alternative approach is the development of multimedia programming and the use of technology to supplement patient understanding. Several studies have demonstrated success in using video-based applications to improve patient knowledge of their health conditions.^4^^,^^13^^,^^14^ However, there is a dearth of research on the use of these models in pathology patient education.

## Materials and methods

### Presentation design

**Fig. 1:**
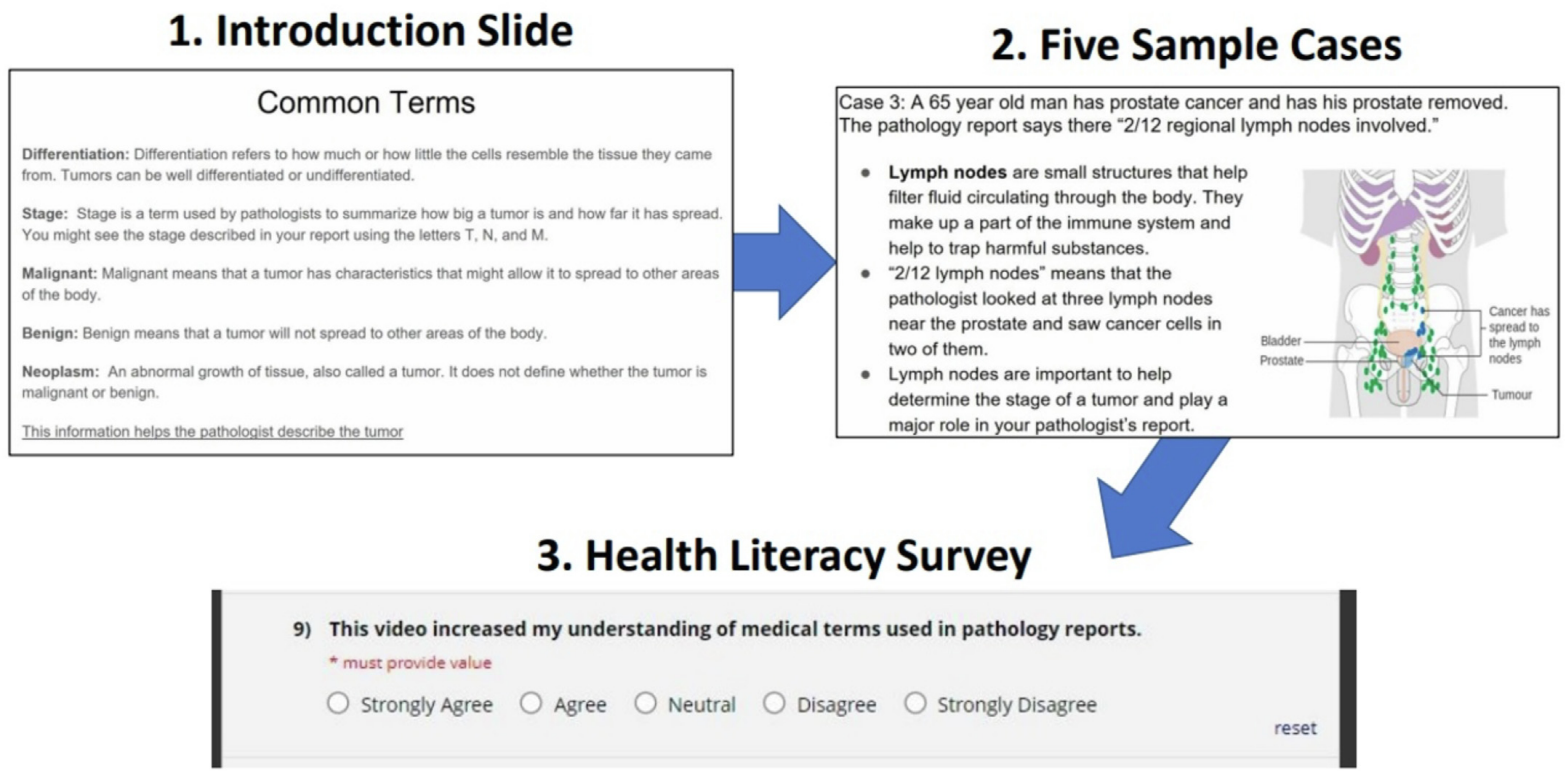
Outline of survey videos. The general progression through each survey video. Respondents were shown one of three videos corresponding to their cancer diagnosis (pancreatic, colorectal, or prostate). Respondents listened to a voiceover concerning common terms they were likely to encounter in their pathology report, followed by five sample cases, and were then asked to compete a brief survey.

Three video presentations were designed to give patients insight into their respective cancer pathology reports and diagnoses: pancreatic, colorectal, and prostatic. Each presentation was approximately 8 min in length and contained five example patient cases with excerpts derived from pathology reports relevant to the specific subtopic: colorectal, pancreatic, or prostatic cancer. A board-certified pathologist and oncologist team assisted with selecting and organizing the material in the cases. For each case, selected medical terminology was drawn from each excerpt and was verbally defined in a brief, bullet point style. The written explanation was evaluated using the Flesch–Kincaid readability tool available in Microsoft Word (Version 2108; Microsoft, Inc.) to ensure that they do not exceed a middle school (grades 6–8 in the United States) reading level. Using the voice record feature of PowerPoint (Version 2108; Microsoft, Inc.), a narrator read each case and provided a more expanded verbal definition of the terms. This is a built-in feature of PowerPoint that presents a slide show and records a user's voice simultaneously. Audio and video files are created once recording is stopped. The videos progressed from a welcome slide to an introduction slide that defined common terms used in pathology reports (e.g., stage, malignant, etc.). Following this, respondents were shown five sequential cases specific to their cancer diagnosis. An example of this progression can be seen in Fig. 1.

Before distribution of the presentation, the authors identified a focus group of ten volunteers, composed of oncology patient care navigators, medical students, and non-medical volunteers, who reviewed and provided feedback on the presentation. The video files were then sent to the volunteers, who were asked to complete a short feedback form (Supplemental Appendix A). They were compensated for their time with a $10 Starbucks gift card. The videos were then embedded in an online survey using the survey tool REDCap (2021, Version 10.6.4).

### Data collection

Patients identified as eligible for this study were approached prior to their appointment at their respective clinic and participating patients signed a consent form within REDCap. Patients then watched their respective diagnosis video in their clinic exam room and completed an electronic survey immediately after watching the entire video. The survey (Supplemental Appendix B) contained demographic questions about the patients' age, gender, level of education, and race/ethnicity, in accordance with the 1997 Office of Management and Budget standards.^15^ Additionally, another question concerning whether the patients used a patient portal to access health information was asked. A question “How confident are you with filling out medical forms by yourself?” was also asked to help to establish baseline health literacy as established by Chew et al.^16^ Finally, five further questions were asked to evaluate participants’ understanding of the role of the pathologist and the health information contained within their medical record concerning their respective diagnosis. The questions were scored using a Likert scale ranging from “strongly disagree” to “strongly agree” with radial buttons for patients to click; there were five possible choices in this scoring system. Two final open-ended questions were asked to allow participants to submit additional inquiries pertaining to the topics covered and suggest topics that they would like featured in future presentations. All survey results were stored within REDCap as de-identified entries.

#### Data analysis

De-identified survey results were exported as Microsoft Excel files from REDCap and subsequent statistical analysis for average Likert scores, standard deviation (SD), and confidence interval (CI, 95%) was carried out using Microsoft Excel 2016 (Version 2108; Microsoft, Inc.); this was accomplished by converting Likert scores to discrete numerical values, with a value of 1.00 for the response “Strongly Disagree” and a value of 5.00 for the response “Strongly Agree.” Average, standard deviation, and confidence interval values came from the five health literacy survey questions asked and the additional question: “How confident are you with filling out medical forms by yourself?” These values were continuous and rounded to two decimal places. Mean multivariate analysis of variance (MANOVA) via Wilkes’ Lambda test with p < 0.05 indicating significance was performed individually for the following independent variables: cancer diagnosis (colorectal, pancreatic, or prostate), self-reported race (White non-Hispanic, African American, or Asian), and self-reported education level (no high school, some high school, some college, four-year college degree, or graduate school). Analysis was performed using JMP v.16 pro (SAS, Inc.). Dependent variables in this analysis were the converted numerical Likert scores for each health literacy-focused survey question (five total questions). No statistically significant difference between groups within each independent variable served as the null hypothesis for this analysis.

## Results

### Study population

Patients over the age of 18 with a primary diagnosis of pancreatic, colorectal, or prostate cancer were identified as they came to their oncology clinic appointment and asked to participate in the study. Exclusion criteria included patients with a work history in the medical field and if a patient had a non-primary diagnosis of either pancreatic, colorectal, or prostatic cancer; patients with more than one of the three surveyed cancers were not allowed to answer the survey multiple times during this study. Thirty-one patients with a primary colorectal cancer diagnosis, 30 patients with a primary prostate cancer diagnosis, and nine patients with a primary pancreatic cancer diagnosis were surveyed. Patient age ranged from 22 to 89, with the average patient age across all cancer diagnoses being about 68 years old. All patients resided in the United States and spoke English as their primary language. Patient self-identified demographics were as follows: 1% Asian, 39% African American, and 60% White; non patients identified as Hispanic or Latino. Fifty-one percent of survey respondents reported having at least some formal college education, while 29% reported having only completed high school. The entire dataset can be found in Supplemental Table 1.

### Assessing change in health literacy

**Fig. 2:**
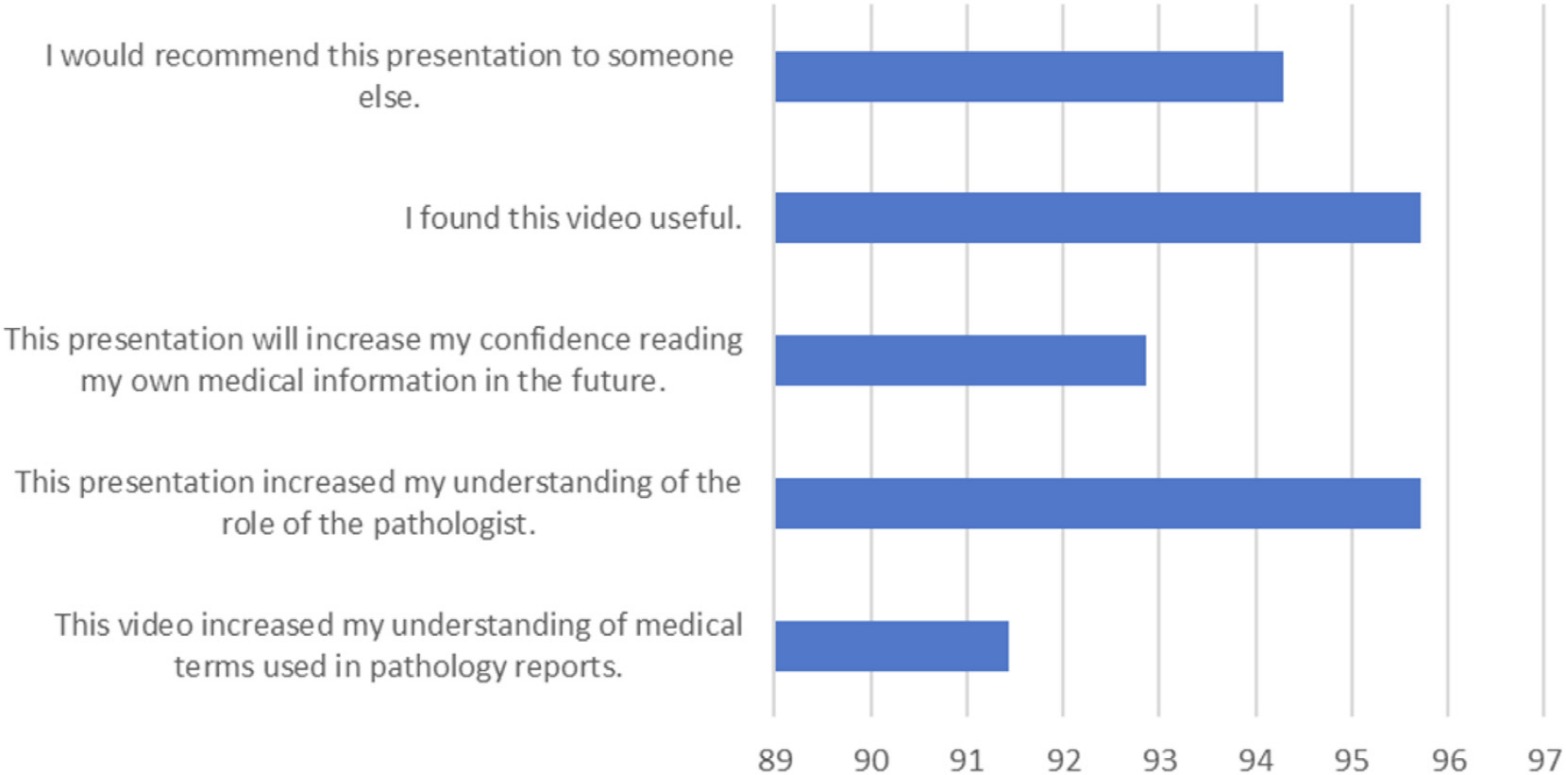
Percentage of patients agreeing or strongly agreeing with survey statements (n = 70). Percentage of respondents agreeing or strongly agreeing (x-axis) with the five survey questions concerning perceived change in health literacy (y-axis).

The first question asked after demographic information on the survey was regarding patients using an online patient portal to access their health information; 58% reported that they did. Ninety-one percent of patients reported they agreed or strongly agreed that the video they watched for their specific cancer diagnosis increased their understanding of the medical terms used in their pathology report(s), with a corresponding average Likert score (ALS) of 4.21 (SD = 0.77, CI = ±0.18); this value lies between the discrete Likert scores of 4.00 and 5.00, with 4.00 corresponding to “Agree” and 5 corresponding to “Strongly Agree.” As the mean score was 4.21, the mean responses lie closer to “Agree” than “Strongly Agree.” The overall percentages of patients either agreeing or strongly agreeing with survey questions can be seen in Fig. 2. All ALS, SD, and CI for the five questions analyzed can be found in Table 1. Ninety-five percent went on to state they either agreed or strongly agreed that the presentation they watched increased their understanding of the role of the pathologist in diagnosing their cancer (ALS = 4.27; SD = 0.65, CI = ±0.15) and reported they found the video useful (ALS = 4.27; SD = 0.53, CI = ±0.13). Ninety-two percent reported they either agreed or strongly agreed that the video they watched increased their confidence in reading their own medical information for the future (ALS = 4.20; SD = 0.71, CI = ±0.17). Finally, 94% of patients reported they agreed or strongly agreed that they would recommend the presentation they viewed to someone else (ALS = 4.29; SD = 0.56, CI = ±0.13).

**Table 1:** Average Likert score (ALS) analysis of pathology health literacy survey results (n = 70).

Question asked	ALS^a^	Standard deviation	95% Confidence interval
This video increased my understanding of medical terms used in pathology reports.	4.21	0.77	0.18
This presentation increased my understanding of the role of the pathologist.	4.27	0.65	0.15
This presentation will increase my confidence reading my own medical information in the future.	4.20	0.71	0.17
I found this video useful.	4.27	0.53	0.13
I would recommend this presentation to someone else.	4.29	0.56	0.13

a A value of 1.00 corresponds to a survey response of “Strongly Disagree” and a value of 5.00 corresponds to a survey response of “Strongly Agree.”

### Confidence in filling out medical forms versus self-reported education level

A breakdown of the survey respondents by their self-reported education level yields the following: 20 respondents reported having only a four-year college degree, four reported having a graduate degree, 16 reported having at only some college education, 20 reported having only a high school degree, and seven reported having only some high school education. The Average Likert Score(s) (ALS) can be found in Table 2; average ALS across all six educational level strata was 3.18 (SD = 0.97, 95% CI = 0.78).

**Table 2:** Self-reported education level versus confidence with filling out medical forms (n = 70).

Self-reported education level	Average Likert score (ALS)^a^
Graduate school (n = 4)	4.25
Four-year college degree (n = 20)	4.30
Some college (n = 16)	3.50
High school (n = 20)	3.20
Some high school (n = 7)	1.86
No high school (n = 3)	2.00

a Responses were to the question: “How confident are you filling out medical forms by yourself.” Likert values were derived by converting each of the five Likert choices to a numerical value: 1.00 for “Strongly Disagree” and 5.00 for “Strongly Agree.” Average ALS across all six educational level strata was 3.18 (SD = 0.97, 95% CI = 0.78).

Analyzing the survey responses to the question: “How confident are you filling out medical forms by yourself” versus the respondents' self-reported education level revealed a linear trend implying decreased self-reported confidence in filling out medical forms coinciding with decreased self-reported education level. Utilizing Excel to create a scatterplot of Table 2 and creating a linear trend line with the equation: ALS = −0.54∗(Self-reported Education Level) + 5.07 yields an R^2^ value of 0.902, signifying relatively good fit. Given the lack of information collected regarding the degrees participants earned or their professions, and the specific amount of education each received (e.g., some college could be one day or three years, graduate school could be a master's degree or a Ph.D., etc.), it is difficult to directly correlate higher levels of self-reported education with increased self-reported confidence filling out medical forms based solely on these data, but such a trend makes some inherent sense.

### Mean Multivariate Analysis of Variance (MANOVA)

Mean MANOVA analysis of Average Likert Scores (ALS) via Wilkes’ Lambda Value (WLV) for the independent variables: cancer diagnosis, self-reported race, and self-reported education level can be found in Table 3. Cancer diagnosis MANOVA yielded a WLV of 0.92 and a p-value of 0.74 and self-reported education level yielded a WLV of 0.64 and a p-value of 0.101, indicating no statistically significant difference was seen among the Likert scores submitted between patients with differing cancer diagnoses or among patients with varying self-reported education levels. Self-reported race MANOVA yielded a WLV of 0.49 and a p-value of <0.001; however, controlling for outliers (there was only one patient that selected “Asian” as their self-reported race) yielded a WLV of 0.03 and a p-value of 0.80, indicating no statistically significant difference was seen among patients that selected “White, non-Hispanic/Latino” or “African American/Black” on the pathology health literacy survey.

**Table 3:** Results from mean multivariate analysis of variance (MANOVA) on cancer diagnosis, self-reported race, and self-reported education level (n = 70).

	Independent variables			
	Cancer diagnosis	Self-reported race		Self-reported education level
		With outliers	Without outliers	
Wilkes' Lambda value (WLV)	0.92	0.49	0.03	0.64
p-value	0.74	0.00^a^	0.80	0.10

a Indicates statistically significant value < 0.05.

## Discussion

### Impact on patient health literacy

Based on the overwhelmingly positive feedback concerning patient survey responses (average Likert Score, or ALS, values all above 4.00, indicating patients on average at least agreed with the questions asked), the implication of this study is that patients both find use with curated videos designed to explain terminology used in pathology reports and believe that watching such videos increases their health literacy. With about six in 10 patients utilizing an online portal to view their health information, a significant portion of patients could benefit from a video specific to their diagnosis (as reflected in their pathology report), perhaps as a link embedded in the report or sent to them by their oncologist. An interesting point seen in this study was the 95% of patients either agreeing or strongly agreeing that the video they watched improved their understanding of the pathologist's role in diagnosing their cancer. Utilizing videos to explain pathology reports could strengthen the doctor–patient relationship by informing patients of the many different medical specialties involved in diagnosing their cancer and could help explain to patients how their diagnosis was ultimately reached. Doing so may ultimately inform patient expectations surrounding their initial diagnosis.

Mean multivariate analysis of variance (MANOVA) data supports the utility of the videos used in this study to patients across multiple cancer diagnoses (colorectal, pancreatic, and prostate), as well as among patients with differing education levels and cultural backgrounds. This is important, as no two patients are the same and ensuring information is conveyed in a way all patients can understand can boost health literacy with regards to cancer diagnosis and treatment. This also provides an interesting context to the results from the analysis of self-reported education level versus ALS. Based on the responses (see Table 2), one might be inclined to think that respondents would report different ALS values for each of the health literacy survey questions regarding perceived changes in health literacy after watching their diagnosis video based on their self-reported education levels. After all, the respondents with greater education levels in general reported greater confidence in filling out their own medical forms. However, because mean MANOVA analysis found no statistically significant difference between respondents based on their self-reported education level, it is implied that the videos shown to survey respondents were at least perceived as increasing health literacy across all self-reported education levels.

### Potential health impacts

Increasing patient health literacy has the potential to improve the oncologist-patient relationship by answering simple questions a patient may have after their initial diagnosis and ultimately streamlining their clinic visits. As discussed earlier, patients who are involved in their care have been found to have better health outcomes.^3^^,^^4^ Increasing patient health literacy using videos specific to a patient's diagnosis could increase patient involvement in their own care, ultimately augmenting patient compliance. Multimedia materials could reduce the educational burden healthcare workers have regarding teaching patients about their health, so long as they are curated with correct, straightforward information. The potential drawback of such methods though, is that they could confuse patients or convey incorrect information.

### Recommendations

Overall, the authors recommend that future work concerning patient health literacy with pathology reports contain detailed stepwise directions on how to read pathology reports and touch on the main parts of the report. For example, for a report on colon cancer, the patient could be clued into understanding the primary assessment and diagnosis, followed by the supporting information and ending with a conclusion from the report. Some of the surgical clinicians involved in this study recommended that the curated videos could be used to explain the common components of pathology reports, such as if synoptic pathology reporting is utilized. This format has been found to result in improved reporting of relevant clinical data.^17^ Furthermore, future videos of the nature used in this project will benefit greatly from having clinicians in both medical/surgical oncology and pathology review materials. Future studies could utilize testing to determine if curated videos increase patient health literacy (such as with pre- and post-quizzes for patients to answer). Creating a Frequently Asked Questions (FAQ) list for patients could also be useful. Finally, future videos/surveys may benefit from having patients be sent a link via SMS or email, so that they may watch the video in the comfort of their own home.

### Limitations

The authors acknowledge the videos used in this project are of limited use for patients that have difficulty accessing online material or that may have hearing loss. Additionally, patients who have been undergoing treatment for cancer for many years may already have the knowledge base that the videos are presenting and thus may not find the videos as useful. Furthermore, having patients watch videos and answer surveys in-clinic ensured patients watched each video from start to finish and correctly completed the survey; however, using this protocol is labor-intensive and has the potential to influence patient responses.

## Data sharing and availability

The datasets generated for this study are available from the corresponding author upon reasonable request.

## Ethics

The studies involving human participants were reviewed and approved by the East Carolina University and Medical Center Internal Review Board (UMCIRB) prior to the start and can be found under identification number 19–001320 and title: “Pathology health literacy.” This IRB is active at the time of submission. Written informed consent for participation was obtained electronically for this study in accordance with the national legislation and the institutional requirements.

## Author contributions

AT Khanchandani and MC Larkins split data collection, data analysis, and paper writing equally. AM Tooley facilitated video design and set up the electronic survey for this project. DB Meyer assisted with minor data analysis. V Chaudhary facilitated the project workflow and initial data collection. JT Fallon served as the faculty investigator for this project.

## Funding

The article processing fee for this article was funded by an Open Access Award given by the Society of ‘67, which supports the mission of the Association of Pathology Chairs to produce the next generation of outstanding investigators and educational scholars in the field of pathology. This award helps to promote the publication of high-quality original scholarship in *Academic Pathology* by authors at an early stage of academic development. AM Tooley received funding from the APC Society of ‘67 Pathology Trainee Project Grant in Health Services Research and Education and the Brody School of Medicine Summer Scholars Program in 2018.

## Declaration of competing interest

The authors declare that the research was conducted in the absence of any commercial or financial relationships that could be construed as a potential conflict of interest.

## References

[1] Kadakia K.T., Howell M.D., DeSalvo K.B. (2021). Modernizing public health data systems. JAMA.

[2] American Medical Association (2013). https://policysearch.ama-assn.org/policyfinder/detail/health%20literacy?uri=%2FAMADoc%2FHOD.xml-0-746.xml.

[3] Regeer H., van Empelen P., Bilo H.J.G., de Koning Ejp, Huisman S.D. (2021). Change is possible: how increased patient activation is associated with favorable changes in well-being, self-management and health outcomes among people with type 2 diabetes mellitus: a prospective longitudinal study. Patient Educ Counsel.

[4] Allen L.A., Venechuk G., McIlvennan C.K. (2021). An electronically delivered patient-activation tool for intensification of medications for chronic heart failure with reduced ejection fraction. Circulation.

[5] Mossanen M., True L.D., Wright J.L., Vakar-Lopez F., Lavallee D., Gore J.L. (2014). Surgical pathology and the patient: a systematic review evaluating the primary audience of pathology reports. Hum Pathol.

[6] Austin E.J., Lee J.R., Bergstedt B. (2021). “Help me figure this out”: qualitative explorations of patient experiences with cancer pathology reports. Patient Educ Counsel.

[7] Winget M., Haji-Sheikhi F., Brown-Johnson C. (2016). Electronic release of pathology and radiology results to patients: opinions and experiences of oncologists. J Oncol Pract.

[8] Weiss B.D. (2003). http://www.hhvna.com/files/Courses/HealthLiteracy/Health_Literacy_Manual_AMA_Revised.pdf.

[9] Mossanen M., Calvert J.K., Wright J.L., True L.D., Lin D.W., Gore J.L. (2014). Readability of urologic pathology reports: the need for patient-centered approaches. Urol Oncol: Seminars and Original Investigations.

[10] Kutner M., Paulesn C., Greenberg E. (2006). https://nces.ed.gov/pubs2006/2006483.pdf.

[11] Prabhu A.V., Kim C., Crihalmeanu T. (2017). An online readability analysis of pathology-related patient education articles: an opportunity for pathologists to educate patients. Hum Pathol.

[12] Fiscella J. (2015). https://www.captodayonline.com/introducing-patients-to-their-pathology-reports-0114/.

[13] Wang D.S., Jani A.B., Sesay M. (2014). Video-based educational tool improves patient comprehension of common prostate health terminology. Cancer.

[14] Chiswell M., Smissen A., Ugalde A. (2016). Using webinars for the education of health professionals and people affected by cancer: processes and Evaluation. J Cancer Educ.

[15] Revisions to the Standards for the Classification of Federal Data on Race and Ethnicity. Office Of Management and Budget. Accessed October 2018. https://www.whitehouse.gov/wp-content/uploads/2017/11/Revisions-to-the-Standards-for-the-Classification-of-Federal-Data-on-Race-and-Ethnicity-October30-1997.pdf

[16] Chew L.D., Griffin J.M., Partin M.R. (2008). Validation of screening questions for limited health literacy in a large VA outpatient population. J Gen Intern Med.

[17] Sluijter C.E., van Lonkhuijzen L.R., van Slooten H.-J., Nagtegaal I.D., Overbeek L.I. (2016). The effects of implementing synoptic pathology reporting in cancer diagnosis: a systematic review. Virchows Arch.

