# Supplementary material for: The impact of curated educational videos on pathology health literacy for patients with a pancreatic, colorectal, or prostate cancer diagnosis
Source: Acad Pathol. 2022 Aug 6;9(1):100038. doi: 10.1016/j.acpath.2022.100038 (PMC9379518; doi:10.1016/j.acpath.2022.100038)
Supplement: Multimedia component 1 [file mmc1.pdf]

SUPPLEMENTAL TABLE 1: DATASET

Patient Number (n=70)	Cancer Diagnosis	Self-Reported Race	Self-Reported Education Level:	Question Asked (5 Likert Choices; Lowest Likert = 1, Highest Likert = 5):						
				Do you use a patient portal to access your health information?	How confident are you filling out medical forms by yourself?	This video increased my understanding of medical terms used in pathology reports.	This presentation increased my understanding of the role of the pathologist.	This presentation will increase my confidence reading my own medical information in the future.	I found this video useful.	I would recommend this presentation to someone else.
1	Prostate	White, non-Hispanic/Latino	High School	Yes	Quite a bit	Strongly Agree	Strongly Agree	Strongly Agree	Strongly Agree	Strongly Agree
2	Prostate	White, non-Hispanic/Latino	Some College	Yes	Quite a bit	Strongly Agree	Strongly Agree	Agree	Agree	Agree
3	Prostate	White, non-Hispanic/Latino	Some College	Yes	Extremely	Agree	Agree	Agree	Agree	Agree
4	Prostate	African American/Black	High School	No	Not at all	Disagree	Strongly Agree	Agree	Agree	Agree
5	Prostate	African American/Black	Some College	Yes	Extremely	Agree	Agree	Agree	Agree	Agree
6	Prostate	African American/Black	Some High School	No	Not at all	Strongly Disagree	Strongly Disagree	Agree	Agree	Agree
7	Prostate	White, non-Hispanic/Latino	Graduate School	Yes	A little	Neutral	Agree	Agree	Agree	Agree
8	Prostate	White, non-Hispanic/Latino	Four Year College	Yes	Extremely	Agree	Neutral	Agree	Agree	Agree
9	Prostate	African American/Black	Four Year College	No	Quite a bit	Agree	Agree	Agree	Agree	Agree
10	Prostate	African American/Black	Four Year College	Yes	Extremely	Neutral	Agree	Agree	Agree	Agree
11	Prostate	African American/Black	Some College	Yes	Somewhat	Strongly Agree	Strongly Agree	Strongly Agree	Strongly Agree	Strongly Agree
12	Prostate	African American/Black	No High School	No	Not at all	Strongly Agree	Agree	Agree	Agree	Agree
13	Prostate	African American/Black	Some High School	Yes	Somewhat	Strongly Agree	Strongly Agree	Strongly Agree	Strongly Agree	Strongly Agree
14	Prostate	African American/Black	Some College	No	Somewhat	Agree	Agree	Agree	Agree	Strongly Agree
15	Prostate	African American/Black	High School	Yes	Somewhat	Agree	Agree	Neutral	Agree	Neutral
16	Prostate	African American/Black	Some High School	No	A little	Agree	Agree	Agree	Neutral	Agree
17	Prostate	White, non-Hispanic/Latino	Some College	Yes	Somewhat		Agree	Agree	Agree	Agree
18	Prostate	African American/Black	No High School	No	Quite a bit	Agree	Agree	Agree	Agree	Agree
19	Prostate	White, non-Hispanic/Latino	Four Year College	Yes	Extremely	Agree	Agree	Agree	Agree	Agree
20	Prostate	African American/Black	Some High School	No	A little	Strongly Agree	Strongly Agree	Strongly Agree	Strongly Agree	Strongly Agree
21	Prostate	African American/Black	High School	Yes	Quite a bit	Strongly Agree	Strongly Agree	Strongly Agree	Strongly Agree	Strongly Agree
22	Prostate	White, non-Hispanic/Latino	Some College	Yes	Quite a bit	Strongly Agree	Strongly Agree	Strongly Agree	Strongly Agree	Strongly Agree
23	Prostate	African American/Black	Some College	Yes	Somewhat	Agree	Agree	Agree	Agree	Agree
24	Prostate	African American/Black	High School	No	Somewhat	Agree	Agree	Agree	Agree	Agree
25	Prostate	White, non-Hispanic/Latino	Graduate School	No	Extremely	Strongly Agree	Strongly Agree	Strongly Agree	Strongly Agree	Strongly Agree
26	Prostate	White, non-Hispanic/Latino	High School	Yes	Not at all	Agree	Agree	Disagree	Agree	Agree
27	Prostate	White, non-Hispanic/Latino	Graduate School	Yes	Extremely	Agree	Agree	Agree	Agree	Neutral
28	Prostate	African American/Black	High School	No	A little	Strongly Agree	Strongly Agree	Strongly Agree	Strongly Agree	Strongly Agree
29	Prostate	Asian	Some High School	No	Not at all	Agree	Agree	Strongly Disagree	Agree	Agree
30	Prostate	African American/Black	Four Year College	No	Extremely	Agree	Agree	Agree	Agree	Agree
1	Colon	White, non-Hispanic/Latino	High School	No	Quite a bit	Strongly Agree	Strongly Agree	Strongly Agree	Strongly Agree	Strongly Agree
2	Colon	White, non-Hispanic/Latino	Some College	No	Somewhat	Agree	Agree	Agree	Agree	Agree
3	Colon	African American/Black	Some College	Yes	Somewhat	Strongly Agree	Strongly Agree	Strongly Agree	Strongly Agree	Strongly Agree
4	Colon	White, non-Hispanic/Latino	High School	No	Somewhat	Agree	Agree	Agree	Agree	Agree
5	Colon	African American/Black	Four Year College	Yes	Extremely	Agree	Agree	Agree	Agree	Agree
6	Colon	White, non-Hispanic/Latino	Four Year College	Yes	Quite a bit	Strongly Agree	Agree	Agree	Agree	Agree
7	Colon	White, non-Hispanic/Latino	Four Year College	Yes	Quite a bit	Strongly Agree	Strongly Agree	Strongly Agree	Strongly Agree	Strongly Agree
8	Colon	African American/Black	High School	No	Quite a bit	Strongly Agree	Strongly Agree	Strongly Agree	Strongly Agree	Strongly Agree
9	Colon	White, non-Hispanic/Latino	High School	Yes	Quite a bit	Strongly Agree	Strongly Agree	Strongly Agree	Strongly Agree	Strongly Agree
10	Colon	White, non-Hispanic/Latino	Some College	Yes	Quite a bit	Strongly Agree	Strongly Agree	Strongly Agree	Strongly Agree	Strongly Agree
11	Colon	White, non-Hispanic/Latino	Four Year College	Yes	Extremely	Strongly Agree	Strongly Agree	Strongly Agree	Strongly Agree	Strongly Agree
12	Colon	White, non-Hispanic/Latino	Four Year College	Yes	Quite a bit	Strongly Agree	Strongly Agree	Strongly Agree	Strongly Agree	Strongly Agree
13	Colon	White, non-Hispanic/Latino	Some College	Yes	Somewhat	Strongly Agree	Strongly Agree	Strongly Agree	Strongly Agree	Strongly Agree
14	Colon	African American/Black	Some High School	Yes	Somewhat	Agree	Agree	Agree	Agree	Agree
15	Colon	African American/Black	No High School	No	Not at all	Agree	Agree	Agree	Agree	Agree
16	Colon	African American/Black	Some High School	No	Not at all	Agree	Agree	Agree	Agree	Agree
17	Colon	White, non-Hispanic/Latino	High School	Yes	Extremely	Strongly Agree	Strongly Agree	Strongly Agree	Strongly Agree	Strongly Agree
18	Colon	White, non-Hispanic/Latino	Some College	No	Somewhat	Agree	Agree	Agree	Agree	Agree
19	Colon	White, non-Hispanic/Latino	Four Year College	No	Somewhat	Agree	Agree	Agree	Agree	Agree
20	Colon	African American/Black	Four Year College	Yes	Extremely	Agree	Agree	Agree	Agree	Agree
21	Colon	African American/Black	Four Year College	Yes	Quite a bit	Agree	Agree	Agree	Agree	Agree
22	Colon	African American/Black	Four Year College	Yes	Extremely	Agree	Agree	Agree	Agree	Agree
23	Colon	White, non-Hispanic/Latino	High School	Yes	Quite a bit	Agree	Agree	Neutral	Agree	Agree
24	Colon	White, non-Hispanic/Latino	Four Year College	Yes	Somewhat	Agree	Neutral	Agree	Agree	Agree
25	Colon	White, non-Hispanic/Latino	Four Year College	Yes	Quite a bit	Agree	Agree	Agree	Neutral	Neutral
26	Colon	White, non-Hispanic/Latino	Some College	Yes	Somewhat	Neutral	Agree	Neutral	Agree	Agree
27	Colon	White, non-Hispanic/Latino	Four Year College	Yes	Extremely	Strongly Agree	Strongly Agree	Strongly Agree	Strongly Agree	Strongly Agree
28	Colon	White, non-Hispanic/Latino	Some College	Yes	Quite a bit	Agree	Agree	Agree	Agree	Agree
29	Colon	White, non-Hispanic/Latino	High School	No	A little	Agree	Agree	Agree	Agree	Agree
30	Colon	White, non-Hispanic/Latino	High School	No	Somewhat	Agree	Agree	Agree	Agree	Agree
31	Colon	White, non-Hispanic/Latino	Four Year College	No	Somewhat	Agree	Agree	Agree	Agree	Agree
1	Pancreatic	White, non-Hispanic/Latino	Graduate School	No	Extremely	Disagree	Agree	Agree	Agree	Agree
2	Pancreatic	White, non-Hispanic/Latino	Four Year College	Yes	Quite a bit	Strongly Agree	Strongly Agree	Strongly Agree	Strongly Agree	Strongly Agree
3	Pancreatic	White, non-Hispanic/Latino	High School	No	Somewhat	Agree	Agree	Agree	Agree	Agree
4	Pancreatic	White, non-Hispanic/Latino	Four Year College	Yes	Quite a bit	Agree	Agree	Agree	Agree	Agree
5	Pancreatic	White, non-Hispanic/Latino	High School	Yes	Quite a bit	Agree	Agree	Agree	Agree	Agree
6	Pancreatic	White, non-Hispanic/Latino	High School	No	Somewhat	Agree	Agree	Agree	Neutral	Neutral
7	Pancreatic	White, non-Hispanic/Latino	High School	No	Somewhat	Agree	Agree	Strongly Agree	Strongly Agree	Strongly Agree
8	Pancreatic	White, non-Hispanic/Latino	Some College	Yes	Somewhat	Strongly Agree	Strongly Agree	Agree	Agree	Strongly Agree
9	Pancreatic	African American/Black	High School	Yes	Quite a bit	Strongly Agree	Strongly Agree	Strongly Agree	Strongly Agree	Strongly Agree
