# Supplementary material for: The impact of curated educational videos on pathology health literacy for patients with a pancreatic, colorectal, or prostate cancer diagnosis
Source: Acad Pathol. 2022 Aug 6;9(1):100038. doi: 10.1016/j.acpath.2022.100038 (PMC9379518; doi:10.1016/j.acpath.2022.100038)
Supplement: Multimedia component 2 [file mmc2.docx]

**Supplemental Appendix A**

**Pathology/Oncology Health Literacy Project Feed Back Form**

FEEDBACK FORM- Understanding Your Pathology Report project

1. Is the description contained in the video for each report scientifically/medically correct? If not, please list the specific video and case.

2. Is the language used in the video accessible to patients? If not please list the specific video and case.

3. Are the images appropriately used and representative of each case?

4. Do you feel the PowerPoint was the appropriate length to cover each topic?

5. Do you feel any topics were left out that should have been covered?

Please leave any other comments or feedback below

Thank you for your participation!
