# Supplementary material for: The impact of curated educational videos on pathology health literacy for patients with a pancreatic, colorectal, or prostate cancer diagnosis
Source: Acad Pathol. 2022 Aug 6;9(1):100038. doi: 10.1016/j.acpath.2022.100038 (PMC9379518; doi:10.1016/j.acpath.2022.100038)
Supplement: Multimedia component 3 [file mmc3.docx]

**Supplemental Appendix B**

**Patient Survey**

Your answers to the survey will be kept confidential and will be recorded in a way that they cannot be tied to your identity. Participation is completely voluntary. If you choose not to answer the survey, it will not affect your care in any way. Please do not enter any of your personal health information in the open-ended portion of the survey.

Age:

Gender:

Are you of Hispanic or Latino/a heritage?

-Yes, of Hispanic or Latino/a heritage -No, not of Hispanic or Latino/a heritage

Race (please select any/all choices that apply):

-American Indian or Alaskan Native -Native Hawaiian or other Pacific Islander

-Asian -Black or African American

-White -Other (please specify):

Highest level of education:

-No High School -Some High School

-High School -Some College

-Four Year Degree -Graduate School

Do you work in the medical field?

-Yes -No

Do you use a patient portal to view your health information?

-Yes -No

Have you ever been diagnosed with cancer?

-Yes -No

How confident are you filling out medical forms by yourself?

-Extremely -Quite a bit -Somewhat -A little -Not at all

Please rate your level of agreement with the following statements.

This video increased my understanding of medical terms used in pathology reports.

-Strongly Agree -Agree -Neutral -Disagree -Strongly Disagree

This video increased my understanding of the role of the pathologist.

-Strongly Agree -Agree -Neutral -Disagree -Strongly Disagree

This video will increase my confidence reading my own medical information in the future.

-Strongly Agree -Agree -Neutral -Disagree -Strongly Disagree

I found this video useful.

-Strongly Agree -Agree -Neutral -Disagree -Strongly Disagree

I would recommend this video to someone else.

-Strongly Agree -Agree -Neutral -Disagree -Strongly Disagree

The next two questions are open ended:

Are there any topics that you would like to see presented in a future video?

Was there any information presented that was not well explained in the video?
